# Veliparib with frontline chemotherapy and as maintenance in Japanese women with ovarian cancer: a subanalysis of efficacy, safety, and antiemetic use in the phase 3 VELIA trial

**DOI:** 10.1007/s10147-022-02258-x

**Published:** 2022-12-19

**Authors:** Mika Mizuno, Kimihiko Ito, Hidekatsu Nakai, Hidenori Kato, Shoji Kamiura, Kimio Ushijima, Shoji Nagao, Hirokuni Takano, Masao Okadome, Munetaka Takekuma, Hideki Tokunaga, Satoru Nagase, Daisuke Aoki, Robert L. Coleman, Yasuko Nishimura, Christine K. Ratajczak, Hideyuki Hashiba, Hao Xiong, Noriyuki Katsumata, Takayuki Enomoto, Aikou Okamoto

**Affiliations:** 1 Department of Gynecology, Aichi Cancer Center Hospital, Nagoya-Shi, Aichi, 464-8681 Japan; 2 Department of Obstetrics and Gynecology, Kansai Rosai Hospital, Amagasaki-Shi, Hyogo, 660-8511 Japan; 3 Department of Obstetrics and Gynecology, Kindai University, Faculty of Medicine, Osakasayama-Shi, Osaka, 589-8511 Japan; 4 Division of Gynecologic Oncology, Hokkaido Cancer Center, Sapporo-Shi, Hokkaido, 003-0804 Japan; 5 Department of Gynecology, Osaka International Cancer Institute, Osaka-Shi, Osaka, 541-8567 Japan; 6 Department of Obstetrics and Gynecology, Kurume University Hospital, Kurume-Shi, Fukuoka, 830-0011 Japan; 7 Department of Gynecologic Oncology, Hyogo Cancer Center, Akashi-Shi, Hyogo, 673-8558 Japan; 8 Department of Obstetrics and Gynecology, Jikei University Kashiwa Hospital, Kashiwa-Shi, Chiba, 277-0004 Japan; 9 Gynecology Service, National Hospital Organization (NHO) Kyushu Cancer Center, Fukuoka-Shi, Fukuoka, 811-1395 Japan; 10 Department of Gynecology, Shizuoka Cancer Center, Sunto-Gun, Shizuoka, 411-8777 Japan; 11 Department of Gynecology, Tohoku University Hospital, Sendai-Shi, Miyagi, 980-8574 Japan; 12 Department of Obstetrics Gynecology, Yamagata University, Faculty of Medicine, Yamagata-Shi, Yamagata, 990-9585 Japan; 13 Department of Obstetrics and Gynecology, Keio University School of Medicine, Shinjuku-Ku, Tokyo, 160-8582 Japan; 14 Department of Gynecologic Oncology, US Oncology Research, The Woodlands, TX USA; 15 AbbVie GK, Minato-Ku, Tokyo, 108-0023 Japan; 16 AbbVie Inc., North Chicago, IL USA; 17 Department of Medical Oncology, Nippon Medical School Musashikosugi Hospital, Kawasaki-Shi, Kanagawa, 211-8533 Japan; 18 Japanese Gynecologic Oncology Group, Shinjuku-Ku, Tokyo, 162-0825 Japan; 19 Department of Obstetrics and Gynecology, Niigata University Graduate School of Medical and Dental Sciences, Niigata, 951-8510 Japan; 20 Department of Obstetrics and Gynecology, The Jikei University School of Medicine, Minato-Ku, Tokyo, 105-8461 Japan; 21 Present Address: Faculty of Medicine, Department of Obstetrics and Gynecology, Kagoshima University, 8-35-1, Sakuragaoka, Kagoshima City, 890-8520 Japan

**Keywords:** Ovarian cancer, PARP inhibitor, Veliparib, VELIA, Antiemetics, Japanese

## Abstract

**Background:**

The phase 3 VELIA trial evaluated veliparib with carboplatin/paclitaxel and as maintenance in patients with high-grade serous ovarian carcinoma.

**Methods:**

Patients with previously untreated stage III–IV high-grade serous ovarian carcinoma were randomized 1:1:1 to control (placebo with carboplatin/paclitaxel and placebo maintenance), veliparib-combination-only (veliparib with carboplatin/paclitaxel and placebo maintenance), or veliparib-throughout (veliparib with carboplatin/paclitaxel and veliparib maintenance). Randomization stratification factors included geographic region (Japan versus North America or rest of the world). Primary end point was investigator-assessed median progression-free survival. Efficacy, safety, and pharmacokinetics were evaluated in a subgroup of Japanese patients.

**Results:**

Seventy-eight Japanese patients were randomized to control (*n* = 23), veliparib-combination-only (*n* = 30), and veliparib-throughout (*n* = 25) arms. In the Japanese subgroup, median progression-free survival for veliparib-throughout versus control was 27.4 and 19.1 months (hazard ratio, 0.46; 95% confidence interval, 0.18–1.16; *p* = 0.1 [not significant]). In the veliparib-throughout arm, grade 3/4 leukopenia, neutropenia, and thrombocytopenia rates were higher for Japanese (32%/88%/32%) versus non-Japanese (17%/56%/28%) patients. Grade 3/4 anemia rates were higher in non-Japanese (65%) versus Japanese (48%) patients. Early introduction of olanzapine during veliparib monotherapy maintenance phase may help prevent premature discontinuation of veliparib, via its potent antiemetic efficacy.

**Conclusions:**

Median progression-free survival was numerically longer in Japanese patients in the veliparib-throughout versus control arm, consistent with results in the overall study population. Pharmacokinetics were comparable between Japanese and non-Japanese patients. Data for the subgroup of Japanese patients were not powered to show statistical significance but to guide further investigation.

## Introduction

Ovarian cancer is diagnosed in an estimated 314,000 women worldwide annually accounting for approximately 207,000 deaths [1]. In Japan alone, there were an estimated 13,400 cases in 2020, and an estimated 4,700 deaths [2]. More than 70% of ovarian cancers are diagnosed at an advanced stage and nearly all ovarian tumors are epithelial in origin [3].

High-grade serous ovarian carcinoma is the most common subtype of epithelial ovarian cancer. It is associated with a high frequency of inherited mutations; approximately 50% of tumors have genomic alterations causing deficiencies in homologous recombination repair [4]. The majority of these are in the breast cancer susceptibility genes (*BRCA*)*1* (55%) or *BRCA2* (19%) [5, 6], and ethnic-specific *BRCA* variations have been identified in Asian countries [7]. Tumor cells harboring such mutations are highly sensitive to DNA-damaging agents, such as platinum-based chemotherapy, and to poly(adenosine diphosphate [ADP]-ribose) polymerase (PARP) inhibitors [8–12].

In the past two decades, the standard-of-care first-line treatment for patients with advanced ovarian cancer has been a platinum-based chemotherapy regimen consisting of carboplatin and paclitaxel. Despite initial positive clinical responses to this treatment, many patients develop resistance after the first or subsequent treatment cycles, with up to 70% reporting disease recurrence [13]. Consequently, patients with advanced ovarian cancer have a poor prognosis, indicating a clear unmet medical need for novel therapeutic strategies to improve survival.

PARP inhibitors, such as olaparib, rucaparib, and niraparib, have proven effective as single agents in treating patients with recurrent ovarian cancer and as maintenance therapy in patients who responded to platinum-based therapy [11, 14–18]. The combination of PARP inhibitors and chemotherapy has been challenging, due to hematologic toxicities leading to necessary dose reductions of both agents [19].

Veliparib (formerly ABT-888) is a potent, highly selective oral PARP-1 and -2 inhibitor shown to enhance the activity of DNA-damaging chemotherapy, including platinum agents [20]. Phase 1 and 2 clinical trials in patients with ovarian cancer have demonstrated antitumor activity and tolerability of veliparib both as a single agent and combined with platinum-based chemotherapy [21–23]. Veliparib also has an overall manageable safety profile in Japanese patients [24–26].

The phase 1 study confirming tolerability of veliparib monotherapy in Japanese patients reported nausea and vomiting in almost all patients (93.8% each), resulting in veliparib dose interruption, reduction, or discontinuation in most patients [26]. Therefore, intensive antiemetic use was encouraged during the veliparib monotherapy phase in the VELIA/GOG-3005 trial (NCT02470585), a randomized, international phase 3 trial evaluating veliparib combined with carboplatin/paclitaxel and continued as maintenance in patients with untreated stage III or IV high-grade serous ovarian carcinoma. In the intention-to-treat population, veliparib combined with carboplatin/paclitaxel and continued as maintenance therapy significantly prolonged progression-free survival (23.5 months) compared with carboplatin/paclitaxel alone (17.3 months) (HR, 0.68; 95% CI 0.56–0.83; *P* < 0.001) [27]. Herein, we report safety, efficacy, and pharmacokinetic analyses from Japan subgroup in the VELIA/GOG-3005 study.

## Patients and methods

The study was conducted in accordance with the International Conference on Harmonization, Good Clinical Practice guidelines, regulations governing clinical study conduct, and ethical principles with their origin in the Declaration of Helsinki. The study was approved by the appropriate Institutional Review Board. All patients provided written informed consent before any study procedures were performed.

### Patients

Patient eligibility criteria for this study have been published previously [27]. Briefly, this study enrolled women ≥ 18 years of age with previously untreated, histologically diagnosed International Federation of Gynecology and Obstetrics (FIGO) stage III or IV high-grade serous ovarian carcinoma with an Eastern Cooperative Oncology Group (ECOG) performance status between 0 and 2.

### Study design and treatments

**Fig. 1:**
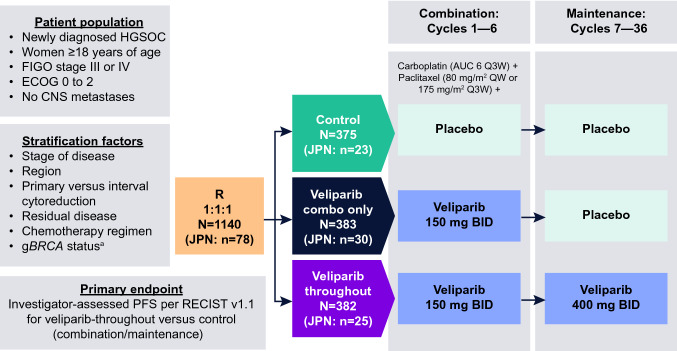
VELIA study design. ^a^Added as stratification factor ~ 14 months after trial initiation, due to noted imbalance. *AUC* area under the curve, *BID* twice daily, *CNS* central nervous system, combo combination, *ECOG* Eastern Cooperative Oncology Group, *FIGO* International Federation of Gynecology and Obstetrics, g*BRCA* clinically significant germline *BRCA*1 or *BRCA*2 mutation, *HGSC* high-grade serous ovarian carcinoma, *JPN* Japan, *OS* overall survival, *PFS* progression-free survival, *Q* every, *R* randomization, *RECIST* Response Evaluation Criteria in Solid Tumors, *W* weeks

The study design is described in Fig. 1. Although frozen tumor sections could be used to provisionally diagnose high-grade serous ovarian carcinoma to proceed with blood draws for germline (g)*BRCA* analysis if written consent was obtained, definitive diagnosis using permanent formalin-fixed paraffin-embedded tumor specimens was required to be eligible for the study. Region of Japan was a stratification factor; other stratification factors have been published previously [27]. Patients were randomized 1:1:1 to one of three study arms: control (placebo with carboplatin/paclitaxel chemotherapy followed by placebo maintenance), veliparib-combination-only (veliparib with carboplatin/paclitaxel chemotherapy followed by placebo maintenance), or veliparib-throughout (veliparib with carboplatin/paclitaxel chemotherapy followed by veliparib maintenance). During the combination therapy phase, patients received oral veliparib (150 mg) or matching placebo twice daily combined with intravenous carboplatin (area under the curve 6 mg/mL/minute every 3 weeks) and paclitaxel (80 mg/m^2^ weekly or 175 mg/m^2^ every 3 weeks) for six 21-day cycles (cycles 1–6). After completion of the combination therapy phase, patients who did not progress per Response Evaluation Criteria in Solid Tumors version 1.1 (RECIST) received oral veliparib (300 mg twice daily, increasing to 400 mg twice daily if tolerated) or matching placebo for an additional thirty 21-day cycles (cycles 7–36) during the maintenance therapy phase.

The primary objective of this study was to compare investigator-assessed progression-free survival for the veliparib-throughout arm versus control arm. As this was the primary objective of the study, data from the veliparib-throughout and control arms are included in this report. Secondary objectives included assessing safety. Pharmacokinetic parameters of veliparib were also evaluated.

### Assessments

Postbaseline tumor assessments were collected at the following intervals: every 9 weeks, the end of the combination phase, every subsequent 12 weeks up to 2 years followed by every 6 months up to 3 years, then annually until disease progression. Plasma samples for pharmacokinetic analyses were collected on day 1 of cycles 1–4.

Treatment-emergent adverse events are defined as adverse events occurring between first dose of veliparib/placebo until 30 days after the last dose. Adverse events were summarized using preferred terms within a System Organ Class according to the Medical Dictionary for Regulatory Activities and graded according to the National Cancer Institute Common Terminology Criteria for Adverse Events version 4.03.

### Statistical analyses

Efficacy analyses were performed on the intention-to-treat population, defined as all randomized patients. Patients who received at least one dose of study drug were included in safety analyses. This manuscript presents data from Japanese patients in the control and veliparib-throughout study arms, because the comparison between these two arms was the primary objective of the overall trial. The cutoff date for data presented in this manuscript was May 3, 2019.

Progression-free survival was estimated using Kaplan–Meier methodology, and progression-free survival was compared between the control and veliparib-throughout treatment arms using the log-rank test. Unless otherwise noted, statistical significance was determined by a one-sided *P* value ≤ 0.025. In accordance with the journal’s guidelines, we will provide our data for the reproducibility of this study in other centers if such is requested.

## Results

### Patient demographics and clinical characteristics

In total, 1140 patients were randomized (control, *n* = 375; veliparib-combination-only, *n* = 383; or veliparib-throughout, *n* = 382). Seventy-eight Japanese patients from 24 institutions in Japan were randomized and received treatment (control, *n* = 23; veliparib-combination-only, *n* = 30; or veliparib-throughout, *n* = 25). Demographics and clinical characteristics are described in Table 1. Compared with non-Japanese patients, a higher proportion of Japanese patients were < 65 years of age, received interval surgery, had ECOG status 0, received every-3-weeks paclitaxel, had any macroscopic disease after primary surgery, and had homologous recombination-deficient tumors.

**Table 1:** Patient demographics and clinical characteristics

Characteristic, *n* (%)	Japanese subgroup		Non-Japanese subgroup	
	Control *n* = 23	Veliparib-throughout *n* = 25	Control *n* = 352	Veliparib-throughout *n* = 357
Median age, years [range]	54.0 [44.0–75.0]	61.0 [43.0–75.0]	62.0 [33.0–86.0]	62.0 [30.0–85.0]
Age group				
< 65	18 (78.3)	17 (68.0)	215 (61.1)	211 (59.1)
≥ 65	5 (21.7)	8 (32.0)	137 (38.9)	146 (40.9)
Median weight, kg [range]	48.1 [39.0–75.3]	53.9 [39.0–74.0]	66.7 [38.1–156.1]	65.7 [39.0–182.0]
Surgery received^a^				
Primary	14 (60.9)	13 (52.0)	236 (67.0)	248 (69.5)
Any macroscopic residual disease after primary surgery, *n*/*N* (%)	6/14 (42.9)	6/13 (46.2)	70/236 (29.7)	77/248 (31.0)
Interval	9 (39.1)	12 (48.0)	98 (27.8)	87 (24.4)
Any macroscopic residual disease after interval surgery^b^, *n*/*N* (%)	3/9 (33.3)	2/12 (16.7)	28/94 (29.8)	25/84 (29.8)
None	0	0	18 (5.1)	22 (6.2)
ECOG performance status^c^, *n*/*N* (%)				
0	19/23 (82.6)	17/25 (68.0)	207/348 (59.5)	207/352 (58.8)
1	4/23 (17.4)	6/25 (24.0)	134/348 (38.5)	135/352 (38.4)
2	0/23 (0.0)	2/25 (8.0)	7/348 (2.0)	10/352 (2.8)
FIGO stage, *n*/*N* (%)				
Stage III	18/23 (78.3)	20/25 (80.0)	274/351 (78.1)	275/357 (77.0)
Stage IV	5/23 (21.7)	5/25 (20.0)	77/351 (21.9)	82/357 (23.0)
Paclitaxel regimen, *n*/*N* (%)				
80 mg/m^2^ every week	6/23 (26.1)	9/25 (36.0)	187/349 (53.6)	181/354 (51.1)
175 mg/m^2^ every 3 weeks	17/23 (73.9)	16/25 (64.0)	162/349 (46.4)	173/354 (48.9)
*BRCA* mutation status^d^, *n*/*N* (%)				
Deleterious mutation	9/23 (39.1)	8/24 (33.3)	83/323 (25.7)	100/329 (30.4)
No deleterious mutation	14/23 (60.9)	16/24 (66.7)	240/323 (74.3)	229/329 (69.6)
Homologous recombination deficiency^e^, *n*/*N* (%)				
Yes	17/23 (73.9)	16/22 (72.7)	190/308 (61.7)	198/317 (62.5)
No	6/23 (26.1)	6/22 (27.3)	118/308 (38.3)	119/317 (37.5)

*BRCA* breast cancer susceptibility gene, *ECOG* Eastern Cooperative Oncology Group, *FIGO* International Federation of Gynecology and Obstetrics

^a^All patients underwent randomization with the intention of undergoing cytoreductive surgery. Some patients did not undergo the planned interval surgery

^b^Data on any residual disease after interval surgery were missing for 4/352 non-Japanese patients in the control arm and 3/357 non-Japanese patients in the veliparib-throughout arm

^c^Data on ECOG performance status scores were missing for 4/352 non-Japanese patients in the control arm and 5/357 non-Japanese patients in the veliparib-throughout arm

^d^Data on *BRCA* mutation status were missing from 1/25 Japanese patients in the veliparib-throughout arm, 29/352 non-Japanese patients in the control arm, and 28/357 non-Japanese patients in the veliparib-throughout arm

^e^A score of ≥ 33 was considered to indicate homologous recombination deficiency status (yes), while a score of < 33 was considered to indicate non-homologous recombination deficiency status (no). Data on homologous recombination deficiency status were missing from 3/25 Japanese patients in the veliparib-throughout arm, 44/352 non-Japanese patients in the control arm, and 40/357 non-Japanese patients in the veliparib-throughout arm

### Concordance between frozen tumor sections and permanent formalin-fixed paraffin-embedded specimens

Fifty-six patients underwent blood draws for g*BRCA* analysis using intraoperative histopathologic diagnosis of high-grade serous ovarian carcinoma, and a definitive diagnosis was available for 55 patients. Of these patients, 52 were diagnosed as high-grade serous ovarian carcinoma (concordance rate: 95%) using permanent formalin-fixed paraffin-embedded specimens. Forty-eight patients were eligible and randomized; the remaining four patients were not enrolled due to reasons other than discordance of histopathologic diagnosis.

### Efficacy

**Fig. 2:**
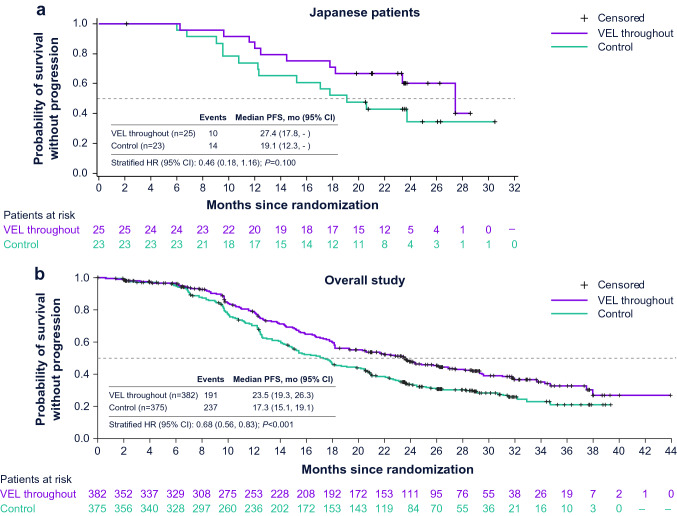
Investigator-assessed progression-free survival in the veliparib-throughout and control groups for Japanese patients and overall study population. Distributions were estimated using Kaplan–Meier methodology in the intention-to-treat populations of Japanese patients (**a**) and the overall study population (**b**). Both graphs present results from the veliparib-throughout arm compared with the control arm (primary end point). Progression-free survival was compared between the control and veliparib-throughout treatment arms using the stratified log-rank test. The dashed line indicates the median, and tick marks indicate censored data. *CI* confidence interval, *HR* hazard ratio, *PFS* progression-free survival, *VEL* veliparib. **b** From *New England Journal of Medicine*, Coleman, R.L., Fleming, G.F., Brady, M.F., Swisher, E.M., Steffensen, K.D., Friedlander, M., Okamoto, A., Moore, K.N., Efrat Ben-Baruch, N., Werner, T.L., Cloven, N.G., Oaknin, A., DiSilvestro, P.A., Morgan, M.A., Nam, J.H., Leath III, C.A., Nicum, S., Hagemann, A.R., Littell, R.D., Cella, D., Baron-Hay, S., Garcia-Donas, J., Mizuno, M., Bell-McGuinn, K., Sullivan, D.M., Bach, B.A., Bhattacharya, S., Ratajczak, C.K., Ansell, P.J., Dinh, M.H., Aghajanian, C., Bookman, M.A., Veliparib with first-line chemotherapy and as maintenance therapy in ovarian cancer, 381, 2403–2415. Copyright © 2022 Massachusetts Medical Society. Reprinted with permission from Massachusetts Medical Society

At the data cutoff for the primary efficacy analysis, progression-free survival in Japan subgroup was longer for the veliparib-throughout arm compared with the control arm (Fig. 2) [27]. In Japan subgroup, median progression-free survival was 27.4 months in the veliparib-throughout arm compared with 19.1 months in the control arm (HR, 0.46; 95% CI 0.18–1.16; *P* = 0.1 [not significant]). This was comparable with the overall study intention-to-treat population, in which median progression-free survival was 23.5 months in the veliparib-throughout arm compared with 17.3 months in the control arm (HR, 0.68; 95% CI 0.56–0.83; *P* < 0.001) [27].

### Pharmacokinetics

**Fig. 3:**
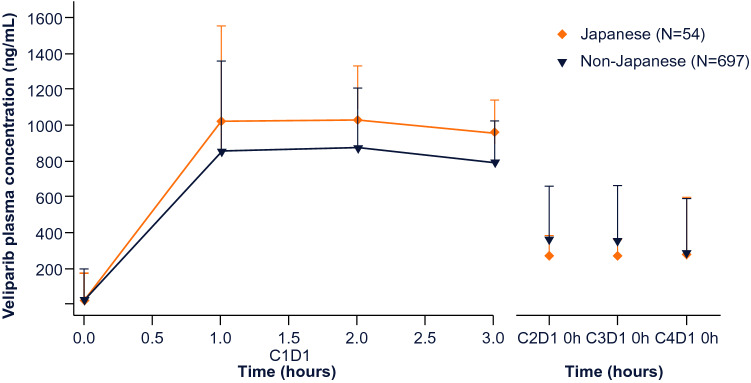
Pharmacokinetics of veliparib in Japanese patients. Veliparib concentrations were measured in plasma samples from Japanese and non-Japanese patients taken on day 1 of treatment for cycles 1–4. Patients in both the veliparib-combination-only and veliparib-throughout arms were included in analyses. *C* cycle, *D* day

Plasma concentrations of veliparib in Japanese and non-Japanese patients are shown in **Fig. ****3**. Veliparib pharmacokinetics were comparable between Japanese and non-Japanese patients at the beginning of these cycles, including patients in both the veliparib-combination-only and veliparib-throughout study arms.

### Safety profile

In both Japanese and non-Japanese subgroups, the median number of cycles of placebo/veliparib received was higher in the control arm compared with the veliparib-throughout arm (Japan, 25 [range, 7–36] versus 17 [2–36]; non-Japan, 18 [1–36] versus 15 [1–36]). The median number of cycles of carboplatin was six for both arms in both subgroups (Japan, range of 6–6 and 4–6 for control and veliparib-throughout, respectively; non-Japan, range of 1–6 for both arms), and the median number of cycles of paclitaxel was six for both arms in both subgroups (Japan, range of 6–6 and 4–6 for control and veliparib-throughout, respectively; non-Japan, range of 1–6 and 1–7, respectively).

An overview of treatment-emergent adverse events is presented in Table 2. The most common adverse events in the veliparib-throughout arm for both Japan and non-Japan subgroups were nausea (100% and 79%) and neutropenia (100% and 74%). In both subgroups, adverse events of nausea were predominantly of grade 1/2 and the most common grade 3/4 adverse events were hematologic.

**Table 2:** Overview of treatment-emergent adverse events by MedDRA System Organ Class and preferred terms

Adverse events, *n* (%)	Japan subgroup				Non-Japan subgroup			
	Control *n* = 23		Veliparib-throughout *n* = 25		Control *n* = 348		Veliparib-throughout *n* = 352	
	Any grade	Grade 3 +	Any grade	Grade 3 +	Any grade	Grade 3 +	Any grade	Grade 3 +
Any	23 (100)	20 (87)	25 (100)	23 (92)	348 (100)	265 (76)	352 (100)	309 (88)
Serious	6 (26)		10 (40)		135 (39)		131 (37)	
Leading to veliparib/placebo discontinuation	0 (0)		5 (20)		43 (12)		92 (26)	
Related to disease progression	0 (0)		0 (0)		18 (5)		6 (2)	
Not related to disease progression	0 (0)		5 (20)		25 (7)		86 (24)	
Leading to veliparib/placebo dose interruption/reduction	5 (22)		17 (68)		174 (50)		259 (74)	
Hematologic								
Anemia	10 (44)	6 (26)	12 (48)	7 (28)	185 (53)	91 (26)	228 (65)	137 (39)
Leukopenia	6 (26)	5 (22)	9 (36)	8 (32)	83 (24)	29 (8)	103 (29)	58 (17)
Neutropenia	21 (91)	19 (83)	25 (100)	22 (88)	230 (66)	164 (47)	259 (74)	196 (56)
Thrombocytopenia	7 (30)	2 (9)	20 (80)	8 (32)	115 (33)	28 (8)	199 (57)	97 (28)
Gastrointestinal								
Constipation	8 (35)	0 (0)	9 (36)	0 (0)	152 (44)	2 (1)	156 (44)	2 (1)
Diarrhea	9 (39)	1 (4)	8 (32)	0 (0)	143 (41)	8 (2)	158 (45)	8 (2)
Nausea	17 (74)	1 (4)	25 (100)	2 (8)	234 (67)	9 (3)	277 (79)	29 (8)
Stomatitis	11 (48)	0 (0)	7 (28)	0 (0)	40 (11)	1 (0)	52 (15)	0 (0)
Vomiting	12 (52)	1 (4)	14 (56)	0 (0)	120 (34)	8 (2)	172 (49)	15 (4)
Other								
Alopecia	18 (78)	0 (0)	17 (68)	0 (0)	197 (57)	2 (1)	180 (51)	0 (0)
Arthralgia	9 (39)	0 (0)	7 (28)	0 (0)	114 (33)	4 (1)	99 (28)	4 (1)
Decreased appetite	8 (35)	0 (0)	7 (28)	0 (0)	77 (22)	3 (1)	104 (30)	7 (2)
Dysgeusia	6 (26)	0 (0)	6 (24)	0 (0)	67 (19)	0 (0)	83 (24)	0 (0)
Malaise	11 (48)	0 (0)	12 (48)	0 (0)	10 (3)	0 (0)	19 (5)	0 (0)
Myalgia	3 (13)	0 (0)	7 (28)	0 (0)	72 (21)	4 (1)	62 (18)	2 (1)
Nasopharyngitis	7 (30)	0 (0)	6 (24)	0 (0)	15 (4)	0 (0)	19 (5)	0 (0)
Peripheral sensory neuropathy	20 (87)	0 (0)	15 (60)	0 (0)	236 (68)	9 (3)	227 (64)	9 (3)

Data include adverse events of any grade that occurred in ≥ 25% of the safety population (e.g., those who received at least one dose of study drug) of Japanese patients in either treatment arm and corresponding adverse events of grade 3 + . Adverse events were reported using preferred terms within a System Organ Class according to the Medical Dictionary for Regulatory Activities (MedDRA) and graded according to the National Cancer Institute Common Terminology Criteria for Adverse Events version 4.03

Common any grade adverse events with ≥ 10% higher rate in the veliparib-throughout versus control arm were nausea and thrombocytopenia (Japan and non-Japan subgroups), and anemia and vomiting (non-Japan subgroup only). Rates of alopecia and of peripheral sensory neuropathy were ≥ 10% higher in the control arm versus the veliparib-throughout arm in Japan subgroup only.

In the veliparib-throughout arm, rates of any grade alopecia, nausea, neutropenia, and thrombocytopenia were > 10% higher in the Japanese subgroup compared with non-Japanese subgroup. Rates of grade 3/4 leukopenia and neutropenia were > 10% higher in the veliparib-throughout arm in the Japanese subgroup compared with the non-Japanese subgroup. Conversely, rates of any grade and grade 3/4 anemia were > 10% higher in the veliparib-throughout arm in the non-Japanese subgroup compared with the Japanese subgroup.

The rate of treatment-emergent adverse events leading to veliparib discontinuation were comparable for patients in the veliparib-throughout arm in the Japanese (*n* = 5, 20%) and the non-Japanese (*n* = 92, 26%) subgroups. Nausea most frequently led to discontinuation among patients in the Japanese subgroup in the veliparib-throughout arm (*n* = 4, 16%). The rate of treatment-emergent adverse events leading to veliparib dose interruption and/or reduction was comparable for the veliparib-throughout arm in the Japanese (*n* = 17, 68%) and the non-Japanese (*n* = 259, 74%) subgroups. The most frequently reported treatment-emergent adverse events leading to veliparib interruption/reduction (≥ 20% of patients) for patients receiving veliparib-throughout in the Japanese subgroup were nausea (*n* = 7, 28%), neutropenia (*n* = 6, 24%), and thrombocytopenia (*n* = 5, 20%).

Rates of gastrointestinal and hematologic adverse events of special interest by treatment phase are described in Table 3. Anemia, neutropenia, thrombocytopenia, and nausea occurred at higher frequencies during combination therapy compared with maintenance in both the Japanese and the non-Japanese subgroups for patients receiving veliparib-throughout. Vomiting occurred at similar frequencies between phases in the non-Japanese subgroup and at a higher frequency during the maintenance phase (*n* = 10, 45%) than the combination therapy phase (*n* = 8, 32%) in the Japanese subgroup.

**Table 3:** Adverse events of special interest by treatment phase

Adverse events of special interest, *n* (%)	Japan subgroup				Non-Japan subgroup			
	Control		Veliparib-throughout		Control		Veliparib-throughout	
	Combination Cycle 1–6 *n* = 23	Maintenance Cycle 7–36 *n* = 23	Combination Cycle 1–6 *n* = 25	Maintenance Cycle 7–36 *n* = 22	Combination Cycle 1–6 *n* = 348	Maintenance Cycle 7–36 *n* = 288	Combination Cycle 1–6 *n* = 352	Maintenance Cycle 7–36 *n* = 288
Hematologic								
Anemia	10 (43)	4 (17)	11 (44)	1 (5)	179 (51)	26 (9)	221 (63)	50 (17)
Neutropenia	21 (91)	8 (35)	24 (96)	9 (41)	226 (65)	30 (10)	255 (72)	42 (15)
Thrombocytopenia	6 (26)	2 (9)	20 (80)	4 (18)	109 (31)	14 (5)	197 (56)	57 (20)
Nonhematologic								
Nausea	14 (61)	7 (30)	21 (84)	14 (64)	211 (61)	69 (24)	223 (63)	158 (55)
Vomiting	9 (39)	7 (30)	8 (32)	10 (45)	104 (30)	30 (10)	123 (35)	95 (33)

Data include adverse events of special interest that occurred in ≥ 25% of the safety population (e.g., those who received at least one dose of study drug) of Japanese patients in either treatment arm. Adverse events were reported using preferred terms within a System Organ Class according to the Medical Dictionary for Regulatory Activities (MedDRA) and graded according to the National Cancer Institute Common Terminology Criteria for Adverse Events version 4.03

For nausea and/or vomiting, antiemetics were used to prevent premature discontinuation of veliparib or to maintain quality of life. At the time of the interim database lock, metoclopramide (68%), olanzapine (56%), prochlorperazine (44%), and granisetron (40%) were frequently administered during veliparib monotherapy maintenance phase for Japanese patients in the veliparib-throughout arm (Table 4). In this arm, 15 (60%) patients received antiemetics on the day of initiation of veliparib maintenance therapy. Four patients prematurely discontinued veliparib due to nausea. Interestingly, all four patients received metoclopramide (single agent: *n* = 2, combination with prochlorperazine or granisetron: *n* = 2). None of the six patients receiving olanzapine on the first day of veliparib maintenance therapy prematurely discontinued veliparib due to nausea and/or vomiting.

**Table 4:** Antiemetic use during maintenance phase in Japan subgroup of veliparib-throughout arm

Antiemetics, *n* (%)	Total (*N* = 25)
Any	21 (84)
Metoclopramide	17 (68)
Olanzapine	14 (56)
Prochlorperazine	11 (44)
Granisetron	10 (40)
Ramosetron	4 (16)
Alprazolam	2 (8)
Lorazepam	2 (8)
Dexamethasone	1 (4)
Domperidone	1 (4)
Lansoprazole	1 (4)
Ondansetron	1 (4)
Travelmin	1 (4)
Vonoprazan	1 (4)

There were no treatment-emergent adverse events leading to death in either treatment arm in the Japanese subgroup. In the non-Japanese subgroup, eight patients (2.3%) in the veliparib-throughout arm and six patients (1.7%) in the control arm had a treatment-emergent adverse event leading to death; none were considered related to veliparib/placebo by the investigator.

## Discussion

In the subgroup analysis presented here, Japanese patients with high-grade serous ovarian carcinoma who received veliparib combined with platinum-based chemotherapy and continued as maintenance experienced numerically longer progression-free survival compared with Japanese patients who received platinum-based chemotherapy alone. This was consistent with the previously published primary analysis of this trial [27]. It is noteworthy that subgroup analysis of Japanese patients was performed to guide further investigation, as it was not powered to show statistical significance.

As g*BRCA* status was required for randomization, it was a significant problem for patients to await initiation of chemotherapy for approximately 2 weeks until g*BRCA* results were available. Provisional pathologic diagnosis using frozen tumor sections takes only several hours from biopsy to diagnosis, with a high concordance rate between rapid and definitive diagnoses [28, 29]. Rapid diagnosis for g*BRCA* blood sampling allows definitive diagnosis and g*BRCA* testing to be performed in parallel, enabling earlier initiation of study treatment.

The most common adverse events observed in Japanese and non-Japanese patients in the veliparib-throughout arm were nausea (100% and 79%, respectively) and neutropenia (100% and 74%), and these adverse events occurred at greater frequencies than in the respective control arms (nausea: 74% and 67%, neutropenia: 91% and 66%). The toxicity profile observed in the veliparib-throughout arm for the Japanese subgroup is comparable with that observed in small phase 1 studies of veliparib as a single agent or combined with carboplatin/paclitaxel in patients with ovarian cancer or other solid tumors [25, 26]. The toxicity profile for the veliparib-throughout arm was generally consistent between the Japanese and non-Japanese subgroups, although frequency of some common adverse events differed between the two subgroups. Specifically, higher rates of alopecia, nausea, neutropenia, and thrombocytopenia were observed in the Japanese subgroup and higher rates of anemia were observed in the non-Japanese subgroup. Utilization of the weekly paclitaxel regimen was more common in non-Japanese (53.3%) than Japanese (31.3%) subgroups in the VELIA study. In the JGOG 3016 study, anemia was more commonly observed for the weekly paclitaxel regimen (69%) than the tri-weekly regimen (44%), with *P* value < 0.0001 [30]. Differences in the utilization of the weekly paclitaxel regimen might be one of the reasons that explains lower incidence of anemia for the Japanese subgroup.

Although pharmacokinetic drug profiles are known to differ among patients of different ethnicities [31], the results of this study demonstrate similar veliparib plasma levels in Japanese patients compared with non-Japanese patients. Notably, this is despite the lower median weight of Japanese patients compared with non-Japanese patients in both the control and veliparib-throughout arms. The similar profiles between subgroups observed here are in line with previous studies [24, 26, 32, 33]. A prior study has also shown no significant pharmacokinetic interaction between veliparib and carboplatin or paclitaxel in Japanese patients [25].

With the comparable veliparib pharmacokinetic profiles in Japanese and non-Japanese patients, the reason for observed differences in frequency of some adverse events between the populations is unclear. Different polymorphisms between populations may lead to differential sensitivity to drug. In a previously reported study, it was hypothesized that the differences in allelic distribution in genes involved in paclitaxel metabolism or DNA repair between Japanese and Western populations may result in differential sensitivity to paclitaxel. However, no significant associations were identified between adverse event frequency and the polymorphisms explored [34]. In the current study, the prevalence of *BRCA* mutations is numerically higher among Japanese vs non-Japanese subgroups. Some reports have indicated increased hematologic toxicity in *BRCA* mutation carriers; however, a recently reported subanalysis of the VELIA trial showed that g*BRCA* status did not impact safety in this study [35]. Additional research into the mechanism underlying the observed differences is needed.

The present study assesses the efficacy and safety of PARP inhibitor therapy during combination chemotherapy and continued as maintenance in patients with newly diagnosed ovarian cancer. Other studies in newly diagnosed patients have generally evaluated PARP inhibitors as maintenance therapy after combination chemotherapy only [18, 36, 37]. Patients enrolled in the current study represent a broad population of patients with advanced high-grade serous ovarian carcinoma who were not required to have a prior response to first-line chemotherapy.

Although the analysis of progression-free survival in Japan subgroup was prespecified, it was not powered for statistical significance and is limited by the relatively small number of patients. While baseline demographic and disease characteristics were generally balanced between treatment arms within the larger non-Japanese subgroup, there were some imbalances within the Japanese subgroup.

Conclusions drawn on differences in rates of specific adverse event rates between Japanese and non-Japanese subgroups are limited by the relatively small number of patients in the Japanese subgroup. Despite the small numbers in the Japanese subgroup, there are consistencies between the observations in this study and in other previous reports. For example, the higher rate of neutropenia observed in the Japanese subgroup vs the non-Japanese subgroup is consistent with a previous report indicating higher rates of grade 3/4 neutropenia for Japanese vs American patients with non-small cell lung cancer receiving carboplatin and paclitaxel [34].

Although nausea was common, it was predominantly low-grade. Nausea was also reported less frequently during the maintenance phase than the combination phase in both Japanese (maintenance: *n* = 14, 64%; combination: *n* = 21, 84%) and non-Japanese (maintenance: *n* = 158, 55%; combination: *n* = 223, 63%) subgroups in the veliparib-throughout arm. PARP inhibitors including olaparib, niraparib, and rucaparib are categorized as moderate to high emetic risk [38]. With such oral anticancer agents, anticipatory nausea and vomiting are quite problematic for patients and may impede their ability to continue taking medication. In these situations, patients sometimes discontinue medication with or without consulting a treating physician. In a phase 1 study of single-agent veliparib in Japanese patients, some patients were treated with olanzapine several days (or weeks) after developing veliparib-induced nausea; however, it was not well-controlled. The NCCN guidelines for antiemesis state that “prevention is key” for anticipatory emesis. Therefore, prophylactic or early antiemetic use including olanzapine was encouraged for Japanese investigators. As seen in Table 4, metoclopramide was the most commonly used antiemetic. However, in previous literature, olanzapine was significantly better than metoclopramide in patients receiving highly emetogenic chemotherapy [39]. Considering these data there are two important points in controlling veliparib-induced nausea and vomiting: 1) early introduction of antiemetics for preventing the initial episode of nausea and/or vomiting, and 2) use of stronger antiemetics such as olanzapine. Although data are limited, early introduction of olanzapine may be effective in controlling veliparib-induced nausea. Optimal strategies for managing nausea and vomiting during treatment warrant further investigation.

### Conclusions

Veliparib combined with chemotherapy followed by veliparib maintenance therapy provided numerically longer progression-free survival compared with chemotherapy alone, with a generally manageable safety profile in Japanese patients with high-grade serous ovarian carcinoma. These data support the efficacy and safety of veliparib combined with platinum-based chemotherapy and continued as maintenance in this population at the dose administered to the global population.

## Data Availability

AbbVie is committed to responsible data sharing regarding the clinical trials we sponsor. This includes access to anonymized, individual and trial-level data (analysis data sets), as well as other information (e.g., protocols and Clinical Study Reports), as long as the trials are not part of an ongoing or planned regulatory submission. This includes requests for clinical trial data for unlicensed products and indications. These clinical trial data can be requested by any qualified researchers who engage in rigorous, independent scientific research, and will be provided following review and approval of a research proposal and Statistical Analysis Plan (SAP) and execution of a Data Sharing Agreement (DSA). Data requests can be submitted at any time and the data will be accessible for 12 months, with possible extensions considered. For more information on the process, or to submit a request, visit the following link: https://www.abbvie.com/our-science/clinical-trials/clinical-trials-data-and-information-sharing/data-and-information-sharing-with-qualified-researchers.html.
